# The Role of Dysregulated Glucose Metabolism in Epithelial Ovarian Cancer

**DOI:** 10.1155/2010/514310

**Published:** 2010-02-17

**Authors:** L. D. Kellenberger, J. E. Bruin, J. Greenaway, N. E. Campbell, R. A. Moorehead, A. C. Holloway, J. Petrik

**Affiliations:** ^1^Department of Biomedical Sciences, University of Guelph, Guelph, ON, Canada N1G 2W1; ^2^Department of Obstetrics and Gynecology, McMaster University, Hamilton, ON, Canada L8S 4L8; ^3^CIHR Group in Matrix Dynamics, University of Toronto, Toronto, ON, Canada M5S 3E2

## Abstract

Epithelial ovarian cancer (EOC) is the most lethal gynecologic cancer and also one of the most poorly understood. Other health issues that are affecting women with increasing frequency are obesity and diabetes, which are associated with dysglycemia and increased blood glucose. The Warburg Effect describes the ability of fast-growing cancer cells to preferentially metabolize glucose via anaerobic glycolysis rather than oxidative phosphorylation. Recent epidemiological studies have suggested a role for hyperglycemia in the pathogenesis of a number of cancers. If hyperglycemia contributes to tumour growth and progression, then it is intuitive that antihyperglycemic drugs may also have an important antitumour role. Preliminary reports suggest that these drugs not only reduce available plasma glucose, but also have direct effects on cancer cell viability through modification of molecular energy-sensing pathways. This review investigates the effect that hyperglycemia may have on EOC and the potential of antihyperglycemic drugs as therapeutic adjuncts.

## 1. Introduction

The poor survival statistics of epithelial ovarian cancer (EOC) are mentioned by way of introduction in almost all review literature pertaining to the disease. Unfortunately, in the past forty years there have been only small improvements in overall ovarian cancer survival rates. Specific challenges to the treatment of EOC include the problems of late detection, metastasis within the peritoneal cavity, drug resistance, and cancer recurrence even after initial response to treatment. Up to 90% of EOCs do not have an identified genetic component, and the development of specific and sensitive screening tools has proven elusive [1]. A metabolic approach to the targeted treatment of EOC has the potential to address many of the issues that make this the most deadly gynecologic cancer. 

 In recent years, it has been noticed that the influence of lifestyle, in particular the high-fat Western diet, is associated with the multisite development of cancers. The state of chronic positive energy balance is linked to a cluster of conditions including impaired glucose regulation and insulin resistance, collectively called the metabolic syndrome [2]. Hyperglycemia is a distinguishing feature of over-nutrition and it is believed to be an independent risk factor for cancer development. To provide an idea of the clinical importance of hyperglycemia, it is estimated that the incidence of type two diabetes mellitus (T2DM), a common consequence of the syndrome, will double in many regions in the next fifteen years. However, the burden of T2DM, where as many as one third of individuals are undiagnosed [3], almost certainly underestimates the true incidence of abnormal glucose homeostasis in the population. Given the emerging association between hyperglycemia and cancer, it is conceivable that there will be an increase in the incidence of EOC in the near future. 

 We hypothesize that hyperglycemia provides a nutrient-rich, growth signal-rich environment for epithelial ovarian cancer cells, where tumour formation and growth is encouraged by free radical-induced DNA damage. We address possible cellular mechanisms by which a hyperglycemic environment may increase the rate of development of ovarian tumours, and discuss the implications for metabolically targeted EOC treatments.

## 2. Hyperglycemia and EOC: Epidemiological Evidence

While significant associations have been reported between elevated glucose [4, 5], glycemic load [6], T2DM [2, 7], and a number of cancers, there is little information to support the influence of preexisting hyperglycemia on EOC [8]. However, much of the literature relating cancer and glucose abnormalities comes from clinical or epidemiological studies that were not originally designed to evaluate the effects of hyperglycemia on cancer development [9]. This is a particular limitation when looking at EOC because of its relatively low population incidence. In addition, many of the studies used diabetic status or a single glucose measurement as a proxy for classifying glucose abnormalities, likely underestimating the true hyperglycemic population. The changing profile of insulin status over the course of T2DM [10] probably further obscured any associations and there was poor consideration of confounding variables such as insulin, obesity, medication, and time since diagnosis. 

 The design of these population studies presumed that hyperglycemia was a direct and sufficient cause of ovarian cancer, when it may in fact be more important in the growth promotion of previously transformed cells. In this way, end-point analyses such as case-control or retrospective cohort studies would not be expected to show any association. A more useful consideration may be that of time to tumour development in patients with hyperglycemia. For example, in women already diagnosed with ovarian cancer, high glucose appears to be a poor prognostic factor [11]. A further complication of these studies is that both hyperglycemia and EOC are notoriously quiet diseases in their early stages. This makes it very difficult from a population health standpoint to infer an association, or suggest causality, as the underlying pathologies of both diseases begin and may interact well before diagnosis. 

 Although population-based studies have not been supportive for a role of preexisting hyperglycemia in the development of ovarian cancer, recent basic science still suggests that EOC may be subject to the influence of high blood sugar. The rate of glucose uptake, which increases with increasing extracellular glucose [12], has been linked with tumour aggressiveness [13]. EOC cells are also sensitive to complete glucose deprivation than nontransformed ovarian epithelial cells [14]; thus, they may also be very responsive to hyperglycemia.

## 3. Hyperinsulinemia versus Hyperglycemia

The impact of hyperinsulinemia on cancer has received much more research attention than the impact of hyperglycemia, although the two conditions are very closely related. It is well established that insulin promotes tumour growth. Insulin is mitogenic via its signaling through the insulin receptor and the insulin-like growth factor (IGF) pathways and direct anabolic signaling which is mediated by changes in the insulin receptor (IR) population. Expression of the IR is elevated in EOC, suggesting a tumour-promoting role in this cancer [15]. 

 However, we contend that the specific impact of hyperglycemia on EOC is also an important area of research as abnormalities in glucose metabolism typically underlie hyperinsulinemia. Elevated insulin levels arise as a result of persistent hyperglycemia and peripheral insulin resistance. Thus, although insulin has direct, isolated actions on tumour growth, changes in glucose metabolism predispose changes in insulin signaling. In addition, it is becoming clear that there are insulin-independent mechanisms of glucose action on cancer risk, particularly through energy-sensing pathways and glucotoxic damage.

## 4. Hyperglycemia

### 4.1. Historical Perspective on Hyperglycemia and Cancer

Almost 80 years ago, Dr. Warburg observed that, compared to normal cells, cancer cells show a preference for glycolysis and lactate production over oxidative phosphorylation [16]. Because glycolysis is 18 times less efficient at producing ATP, this glycolytic switch suggests that cancer cells have an inherently high need for glucose. Furthermore, tumours are very active metabolically and require copious amounts of cellular fuel to meet growth demands. Aerobic glycolysis has been successfully exploited in EOC diagnostics in which tumour visualization occurs through the detection of the differential uptake of glucose in cancer cells compared to normal cells [17]. The use of FdG-PET (18-fluoro-2-deoxyglucose positron emission tomography) demonstrates the association between tumour growth and energy availability. 

 Glucose metabolism of tumours was studied extensively starting in the 1950s. Warburg's initial observation was bolstered by evidence that tumours could induce host hypoglycemia in a tumour mass-dependent fashion [18, 19]. In many tumour-bearing animals, there appeared to be host compensation for hypoglycemia at the level of the liver, with increased gluconeogenesis and glycogen mobilization [18]. Local hypoglycemia in the area around the tumour was particularly pronounced [18, 20]. It was found that while tumours had the capacity to take up larger volumes of glucose in mildly hyperglycemic environments they were poor at compensating for low blood glucose by increasing glucose uptake [18, 20]. An important role for the vasculature was identified in hyperglycemic conditions, as tumours were able to increase glucose uptake by increasing glucose transfer across the capillary walls [20]. 

 Following these metabolic observations, a number of groups looked at the growth characteristics of tumours in hyperglycemic environments. It was reported widely that profound hypoinsulinemia usually caused by chemical destruction of pancreatic *β*-cells consistently caused a decrease in tumour growth [19, 21, 22]. The hypoinsulinemia was generally associated with significant hyperglycemia. However, in diabetic animals, combined treatment of both antitumour and antihyperglycemia therapies gave the best tumour-reductive outcome [19]. 

 Although they demonstrated a negative effect of hyperglycemia on tumour development, these early studies have a number of limitations. The large transplantable tumours used were sustainable in vivo only for several weeks. The alloxan used to induce diabetes was toxic and administered systemically, and so may have had effects outside the target endocrine cells within the pancreas. Also, the studies that showed a decrease in tumour mass in the diabetic animals did not report the changes with respect to total animal mass, which is generally smaller in the diabetic animals. 

 The studies also seem to make the assumption that all glucose taken up is immediately metabolized. However, it was noted independently by several groups that glucose uptake was too high to be fully explained by the amount of tumour growth [20, 23]. These results suggest the possibility that cancer cells may be able to store fuel in times of high abundance. Nigam et al. concluded that low glycogen was due to defective glycogen synthesis and reported low activities of key glyconeogenic enzymes phosphoglucomutase and glycogen synthetase as compared to normal tissues [24]. The low tumour glycogen was also linked to abnormally high rates of glycogen breakdown by phosphorylase. A recent article looking at glycogen levels in human colorectal cancer, however, reported that tumour cells actually had higher glycogen content than normal tissue [25]. The authors noted that there was less glycogen in poorly differentiated tumours compared to well-differentiated tumours, suggesting that low glycogen may be an indicator of a poor prognosis. They also found a very clear negative correlation between glycogen level and proliferation index [25]. The little research in this area has been carried out in normoglycemic conditions. It seems likely that, given the high rate of fuel usage in a tumour, at normoglycemic levels, there would be little need for storage as most would be used immediately. This brings up an intriguing question: could hyperfueled conditions favour a storage phenotype in cancer cells? This might explain the low growth rates of tumours in type one diabetic conditions. 

 Glycogen synthase kinase 3*β* (GSK3) phosphorylates and inactivates glycogen synthase, preventing the formation of glycogen. High levels of GSK3 have been implicated in the progression of a number of cancers, including ovarian cancer [26]. GSK3 affects tumour growth through many different mechanisms, including NF-*κ*B and Wnt signaling activation [26]. Although it was not discussed in the literature reviewed here, GSK overexpression may be linked with glycogen storage and proliferation index. In summary, despite a number of investigations, carbohydrate metabolism by tumours is still poorly understood.

### 4.2. Hyperglycemia in EOC

We consider the possible effects of glucose on EOC development to be either “permissive” or “contributing”. Permissive effects are those that alter the energy status of cells, allowing tumour cells greater access to fuel. Contributing effects are those that directly damage protein or DNA in some cancer-promoting way. 

 Persistent elevations in blood sugar occur once hypersecretion of insulin is no longer able to compensate for combined insulin resistance and high glucose levels. The failure of insulin to facilitate glucose entry into cells is evaluated on a continuum, meaning that patients may have significant pathological changes while being in a “prediabetic” state. In fact, by time of diagnosis of T2DM, hyperglycemia has already caused vascular complications in at least 20% of patients [3, 27]. However, poor glycemic control is not solely due to impaired insulin signaling, as glucose has the ability to regulate its own clearance by mass action [12]. Glucose self-regulation is impaired in people with hyperglycemia, leading to a state of glucose resistance [12]. Chronic hyperglycemia downregulates enzymes responsible for glucose metabolism, including those of the energy-sensing AMP-activated protein kinase (AMPK) pathway [28]. This results in fewer glucose transporters translocating to the cell surface, further impeding the cell's ability to take up fuel. Gluconeogenesis also appears to be increased in patients with already elevated blood sugar [29]. Thus, the effects of glucose join insulin resistance in maintaining and exacerbating hyperglycemia. 

### 4.3. Permissive Effects of High Glucose: Energy Excess

It is postulated that where there is energy available tumour cells will have a suitable soil to grow. The biological plausibility of this excess energy hypothesis has been supported by a number of in vitro studies: Yamamoto et al. found that increasing glucose concentration in the culture media of MCF-7 breast cancer cells increased proliferation [30], mediated by an upregulation of cdk2 and cyclin D1 [31]. In a line of choriocarcinoma cells, sustained hyperglycemia was found to stimulate the cell's glucose transport system, increasing glucose uptake rates [32]. In contrast, most nontransformed cells downregulate glucose transport in the presence of hyperglycemia. Studies in human breast cancer xenografts also suggest that the amount of glucose metabolism is not determined by metabolic demand, but rather by substrate availability [33]. Conversely, energy restriction is protective in several cancer models [34]. Together, these findings support the idea that the fuel availability in hyperglycemia may be permissive for cancer growth. 

 In hyperglycemia-induced insulin resistance, the ability of normal cells to access fuel is impaired. The correlation between cancer risk and T2DM suggests that where normal cells fail metabolically cancer cells excel. Mechanistically, this may involve the overexpression of components of the AMPK pathway [35]. It is possible that in hyperglycemia cancer cells are inherently better at responding to the effects of insulin compared to insulin-resistant “normal” cells. In their 2004 paper, Gatenby and Gillies argue that mutations affecting substrate use cannot be early events in carcinogenesis because they would offer no advantage when there are no constraints on fuel availability, which typically arise in a larger tumour mass [13]. While this is true in a normal cellular environment, in hyperglycemia there is a limit on substrate availability because of insulin resistance. Better access to the abundance of extracellular glucose, therefore, confers a selective growth advantage and could be an early marker of tumourigenic potential. 

 If conditions such as dysglycemia and diabetes prove to be involved in EOC initiation as well as promotion, then we propose that the selective pressures of the energy status may be an early event in the formation of EOC tumours. Cells that are best able to survive high glycemic conditions necessarily have a key characteristic of cancer cells, essentially obtaining self-sufficiency in growth signals [36]. Thus, cancers that arise in a hyperglycemic environment may represent an unregulated adaptive survival response. Although there is currently no directly supportive data for this hypothesis, possible mechanisms for this relationship are described in the following sections. 

### 4.4. Contributing Effects of High Glucose: Cellular and Genetic Damage

The consequences of chronic exposure to high glucose tend to be detrimental to cellular function and affect the physiology of the normal ovary [37]. In fact, most long-term diabetic complications (retinopathy, neuropathy, and nephropathy) are consequences of hyperglycemia and cannot be reversed despite glucose normalization [38]. However, this damage might also provide a mutational advantage to some cells by altering cellular proteins or DNA. Cancer development is often thought of in terms of a series of “hits”. The conditions of the tumour microenvironment, many of them determined by an altered metabolic profile, have been shown to contribute to the genetic instability of cancer cells [39], providing the necessary “hits” for a more aggressive tumour. Acidity, hypoxia, and formation of reactive oxygen species may all be enhanced in tumours in a hyperglycemic environment.

#### 4.4.1. Acidic Environment

In tumour cells, high glucose flux through the glycolytic pathway produces large quantities of lactate, resulting in tumour tissue with pH 0.5 units lower than normal tissue [40]. Cancerous cells adapt to this acidification, exhibiting maximal growth at the relatively low pH of about 6.8 [41]. Tumours also have a capacity, similar to working skeletal muscle, to share lactate between hypoxic and nonhypoxic cells, so it is not extruded as a waste product [42]. Despite these survival adaptations, tumour acidity has been shown to impair DNA repair mechanisms [39] and to upregulate angiogenic molecules such as vascular endothelial growth factor (VEGF) and IL-8 in order to enhance lactate clearance [43, 44]. Experimental evidence demonstrates that the acidic environment is supportive of tumourigenesis, increasing resistance to chemotherapy [45], mutation rate [46], and invasion capability [47]. The acid-mediated tumour invasion hypothesis postulates that H^+^ ions from the tumour microenvironment diffuse down their concentration gradient into the surrounding normal tissue [48]. Because the normal cells cannot survive the increase in acidity, the border of malignant tissue is progressively pushed forward. In fact, mathematical modeling has shown that tumour acid production alone can explain patterns of tumour growth [40]. The effects of acidity are particularly important in a hyperglycemic environment because increased glucose flux through tumour cells has been shown to create a large increase in lactate production [33, 49].

#### 4.4.2. Transient Hypoxia

The characteristic microvascular damage caused by hyperglycemia [50] may lead to periods of hypoxia, possibly through a nitric-oxide-mediated mechanism. The bioavailability of the vasodilator is decreased in diabetes [51] as it is scavenged by superoxide radicals to form the highly reactive ${\text{ONOO}}^{\dot{\;}}$ molecule [52]. Transient hypoxia is thought to be one of the strongest pressures for cells to undergo transformation and is a central hypothesis explaining the glycolytic switch [13, 53]. Hypoxic conditions also increase the activity of hypoxia-inducible factor (HIF-1*α*) and VEGF, which are strongly associated with both tumour angiogenesis and EOC tumour aggressiveness [54, 55].

#### 4.4.3. Oxidative Stress

Levels of oxidative stress reflect the ability to balance production and elimination of highly reactive free radicals, which include the family of reactive oxygen species (ROS). Oxidative stress is known to be higher in diabetic patients than in healthy individuals [56], and it is often cited as a unifying theory to explain tissue damage by hyperglycemia [57]. Because ROS can also create DNA damage through a number of mechanisms [58], it has similarly been proposed that carcinogenesis in general is caused by oxidative stress [59]. This stress in ovarian epithelial cells specifically is thought to be a potential initiator of tumourigenesis [60]. Hyperglycemia also causes increased flux of glucose through the aldose-reductase (polyol) pathway, which has been postulated to increase sensitivity to oxidative stress by reducing regeneration of the antioxidant glutathione [50]. While epidemiological studies evaluating antioxidant use in diabetes [52, 61] and ovarian cancer [62] have not been conclusive, preliminary results suggest that this therapeutic avenue is worth further exploration. A recent study of flavonoids with antioxidant effects found that they inhibited cell growth and VEGF expression in ovarian cancer cells [63].

#### 4.4.4. Glycation

Much of the tissue damage and cellular dysfunction associated with hyperglycemia has been attributed to advanced glycation end products (AGEs) created by the nonenzymatic glycation of proteins [64]. While AGE accumulation is a normal part of aging, it occurs at an accelerated rate in diabetes where progressive modifications can lead to irreversible cross-linking, impairing the actions of other molecules [64, 65]. Receptors for AGE (RAGE) mediate many more severe actions and potentiate the cellular response [66]. RAGEs are upregulated by presence of AGE ligands, and AGE-RAGE binding protects the ligands, allowing them to persist in the environment [66]. AGE-RAGE interaction has been shown to stimulate tumour cell growth or invasiveness in pancreatic cancer [67], melanoma [68], and glioma [69], while blocking the RAGE inhibits tumour formation and metastasis [68, 69]. The ovarian surface epithelium may be particularly susceptible to the effects of glycation damage because not only the tissue is well vascularized, but it is also in constant contact with peritoneal fluid, whose glucose content is reflective of blood glucose levels [70]. 

 Mechanistically, AGE-RAGE signaling has been linked to induction of an inflammatory response in the vasculature [71], as well as an increase in matrix metalloproteinases (MMPs)-2 and -9 [66], and may, therefore, play a role in determining tumour invasiveness. Because AGE-RAGE signaling seems to be part of the chronic rather than acute response [66], its contributions to the development of tumour formation are quite plausible. 

 Glucose reactivity in hyperglycemia can also lead to glucose autoxidation, generating hydroxide radicals, and contributing to the burden of oxidative stress [72]. Also, apart from RAGE signaling, glucose moieties on proteins can donate electrons to form hydrogen peroxide, directly activating NF-*κ*B [73, 74] and contributing to an inflammatory response. There is evidence that changes to local tissue can enhance the possibility of tumour spread [75], possibly implicating glucose-induced damage to the peritoneal cavity as a permissive factor for ovarian tumour metastasis [76].

### 4.5. The Role of Glucose Transporters

Glucose is a large, hydrophilic molecule that cannot diffuse through the lipid bilayer of cells on its own, and thus requires specific transporter proteins. Glucose enters cells by facilitated diffusion mainly through glucose transporters (GLUTs), and the activation of GLUT genes is one of the earliest events in oncogenesis [77]. Because GLUTs have a role in glucose sensing and respond to extracellular glucose concentrations, these transporters may be very important in a hyperglycemic environment. GLUT1 in particular is highly expressed in ovarian cancer [78], where tumour status (benign, borderline, or malignant) is correlated with the level of GLUT1 expression [79]. Almost all invasive epithelial carcinomas are positive for GLUT1, independent of stage, grade, or histological subtype [79, 80]. Antibodies to GLUT1 decrease proliferation, induce apoptosis in nonsmall cell lung cancer and breast cancer cell lines, and appear to synergize with a number of chemotherapeutics to enhance their apoptotic effects [81]. 

 Very recently, another class of transporters, sodium/glucose cotransporters (SGLTs), was shown to be associated with the epidermal growth factor receptor (EGFR) in cancer cells [96]. The authors of the study proposed that SGLTs may enhance tumourigenesis by making cells independent of the glucose concentration gradient, allowing them to take up fuel in any situation. This hypothesis is in line with the proposal made here that permissive effects of glucose are cancer causing: removing restrictions on fuel availability seems to enhance tumourigenesis. The EGFR is particularly important in ovarian cancer; it is normally expressed on ovarian surface epithelium and is often overexpressed in EOC. The expression of key glucose transporters in ovarian cancer is summarized in Table 1.

## 5. Inflammation and EOC

In both rats and humans, hyperglycemia has been shown to be a major cause of the systemic inflammatory response [99, 100]. Both oxidative stress [101] and AGE-RAGE [66] signaling are also implicated in promoting systemic inflammation in hyperglycemic environments. 

 Inflammation is thought to be associated with cancer development mechanistically because of rapid cell division, DNA excision and repair, oxidative stress, and high concentrations of cytokines and prostaglandins; all of which are promoters of mutagenesis [102]. Moreover, inflammation has been proposed as a unifying hypothesis for the development of EOC [103]. The high concentrations of circulating growth-promoting and inflammatory cytokines as a result of hyperglycemia may mean that factors, which normally in an autocrine or paracrine fashion [104] are instead coming from the systemic environment and exerting an endocrine effect, potentiate tumour growth. In support of this, animal knockout studies have shown that MMP production by the host may be more important in carcinogenesis than MMP production by tumour cells themselves [105]. 

 Cytokines can affect EOC tumour growth by acting as growth factors, increasing angiogenesis, or an immunomodulatory pathway whereby they prevent cellular recognition and destruction of the tumour. A number of cytokines that are increased as part of systemic inflammation in diabetes also have tumour promoting effects in ovarian cancer [106]. IL-1 and TNF-*α* are thought to increase production of IL-6, which promotes cell attachment and migration [107] and also blocks apoptosis induced by cytotoxic agents [106]. IL-8 and TGF-*β* promote tumour angiogenesis [106]. In addition, although TGF-*β* normally inhibits epithelial cell proliferation [108], repeated exposure to high levels may attenuate the response of cancerous epithelial cells [106]. 

 The inflammatory hypothesis lends itself to testing with a variety of antiinflammatory drugs and indeed early studies show promise. A study evaluating human ovarian tumours in nude mice concluded that cyclooxygenase inhibitors limited tumour growth, in part through an antiangiogenic mechanism [109]. Epidemiologically, patients with chronic aspirin, NSAID, or acetaminophen use have been shown to have a reduced risk of EOC [110]. However, as with antioxidant trials, these observational studies are still preliminary [103].

### 5.1. The Incessant Ovulation Hypothesis

Recently, the inflammation associated with postovulatory follicle repair has received attention as a possible contributor to EOC promotion [103]. The incessant ovulation hypothesis purports that the repeated damage and repair cycles associated with ovulation enhance the possibility for mutagenesis. Incessant ovulation also increases the likelihood that inclusion cysts will form, trapping epithelial cells in the hormone-rich environment of the ovarian stroma [1, 111]. If these trapped cells are inappropriately maintained, they are more likely to transform [111–113]. Wound healing in hyperglycemia is characteristically slow and almost certainly influenced by the effects of inflammation and damage from glycation. Lowered nitric oxide bioavailability in combination with the tissue damage caused by hyperglycemia may be partly responsible [114]. In one study AGE-RAGE blockade decreased expression of inflammatory cytokines and MMPs resulting in normalization of wound closure in a genetic mouse model of diabetes [115]. Taken together, the mutagenic risk and the risk of entrapment in inclusion cysts from repeated ovulations, combined with impaired wound healing, might mean a greater risk for ovarian cancer development in a hyperglycemic environment. This idea provides a possible mechanism by which hyperglycemia may initiate cancer, in addition to playing a role in promotion of EOC from an unrelated transforming event.

## 6. Glucose, Angiogenesis, and Tumour Formation

As hypothesized by Dr. Folkman [116], solid tumours must recruit new blood vessels in order to grow beyond 1-2 mm in size. Most of the tumour vascularization occurs through angiogenesis, which is the development of new blood vessels from preexisting vasculature. The angiogenic process is regulated by a balance between pro- and anti-angiogenic factors and in ovarian cancer there is a concomitant overexpression of proangiogenic factors and an inhibition of anti-angiogenic molecules [117]. There are numerous reports concluding that elevated glucose levels contribute to increased angiogenic processes. Granulosa cell tumours of the ovary have been shown to have increased expression of members of both the glycolytic and angiogenic pathways [118]. Glucose directly increases expression of the potent proangiogenic factor VEGF, which is thought to be the mechanism involved in the vascular complications associated with diabetes (reviewed in [119]). In a similar fashion to tumour cells, endothelial cells that comprise the tumour vasculature also increase their utilization of glucose. Glucose transporter expression is increased in the hypoxic environment associated with most solid tumours [120], and glucose increases survival of both tumour epithelial and endothelial cells [96]. Because increased tumour vascularity is correlated with increased metastatic potential and tumour progression [121, 122], the proangiogenic inflammatory environment of hyperglycemia may also promote carcinogenesis. Unfortunately, inflammation may be self-promoting as increased tumour perfusion can act to further exacerbate the immune response [121]. 

 In addition to the direct effects of glucose, the effects of inflammation are likely mediated by VEGF. Inflammatory mediators upregulate VEGF and VEGF receptors, which are correlated with the clinical outcomes of ovarian cancer patients [123]. For example, NF-*κ*B can promote angiogenesis by activating VEGF and IL-8 [124] and may be central to inflammation-induced tumour growth and progression [125]. MMPs can also stimulate proliferation and release of VEGF [126]. 

 The possible impact of hyperglycemia-related inflammation on cancer suggests that anti-angiogenic molecules such as thrombospondin-1 may be of great benefit in treating diabetic tumours [127]. The relationship between angiogenesis, inflammation, and carcinogenesis is illustrated by the fact that a number of anti-angiogenic drugs that are promising in the treatment of cancer are also effective against chronic inflammatory diseases [128].

## 7. Antidiabetic Drugs as Targeted EOC Therapy

Because of the multitude of protumour effects of glucose, it is intuitive that glucose deprivation may be a potent antitumour treatment approach. From the literature, it is apparent that glucose is an important energy substrate, survival factor, and proangiogenic molecule. There are a number of antihyperglycemic treatments currently available for reducing serum blood glucose and these drugs may effectively inhibit glucose availability to the tumour. Although the effects of antihyperglycemic drugs are well documented in diabetes, their effects in cancer are relatively unknown. Preliminary reports show that these drugs may have multi-modal effects in slowing tumour growth. In an approach similar to that using anti-angiogenic drugs, the class of antihyperglycemic drugs such as metformin and rosiglitazone may reduce glucose availability to the tumour and essentially starve the tumour of nutrients. These drugs have also been shown to have direct effects on metabolic and signaling pathways that may be independent of glucose. 

 Metformin is in the biguanide class of antidiabetic drugs and decreases circulating glucose levels by suppressing hepatic production of glucose [129]. Metformin, by reducing insulin and glucose levels, reduced the size and increased latency of mammary adenocarcinomas in HER-2/neu transgenic mice, demonstrating a potent antitumour effect [130]. In vitro, metformin significantly inhibits the growth of epithelial ovarian cancer cells and may potentiate the effects of the common chemotherapy drug cisplatin [131]. Metformin may preferentially increase peripheral glucose uptake in skeletal muscle, as administration increases AMPK activity in skeletal muscle [132] and stimulates translocation of muscle GLUT-4 [133]. This favoured packaging of glucose into skeletal muscle cells would decrease serum glucose levels and availability to the tumour cells resulting in nutrient depletion. Stimulation of AMPK by metformin also contributes to the reduced hepatocyte production of glucose [134]. In fact, AMPK activation is associated with an inhibition of tumourigenesis through apoptosis induction, decreased cell proliferation and may be a communal molecule utilized by metformin as well as a number of anti-tumour drugs that have been shown to have effects in EOC. C93 [135], resveratrol [13, 136], 2-deoxy-D-glucose [137], and AICAR [138] are targeted therapies that are effective in the treatment of ovarian cancer. Interestingly, these molecules also cause the stimulation of AMPK, indicating a common pathway intersection with metformin. Although not yet investigated, there is a possibility that metformin may have a synergistic interaction with these molecules, in addition to its glucose deprivation effects. 

 Rosiglitazone is another antidiabetic agent in the thiazolidinedione class of drugs designed to reduce the hyperglycemia associated with this disease. Rosiglitazone activates the peroxisome proliferator activated receptors (PPAR) in target tissues, increasing insulin sensitivity and decreasing serum levels of glucose. As with metformin, rosiglitazone also stimulates increased expression of GLUT-4 [139] causing glucose uptake in skeletal muscle [140]. One of the mechanisms by which rosiglitazone may have a significant antitumour effect is through the inhibition of angiogenesis. Rosiglitazone has been shown to inhibit VEGF-induced angiogenesis [141] and is suggested as a treatment option for vascular disorders associated with diabetes such as diabetic retinopathy, macular degeneration, and so forth. As VEGF expression is significantly elevated in EOC [142] and is responsible for some of the ovarian tumour vascularization (reviewed in [143]), rosiglitazone may have a bimodal anti-tumour effect by decreasing glucose availability and also by reducing tumour angiogenesis. Simply by decreasing tumour vascularity, rosiglitazone will decrease glucose delivery to the tumour by decreasing tumour tissue perfusion.

## 8. Summary and Conclusions

**Figure 1:**
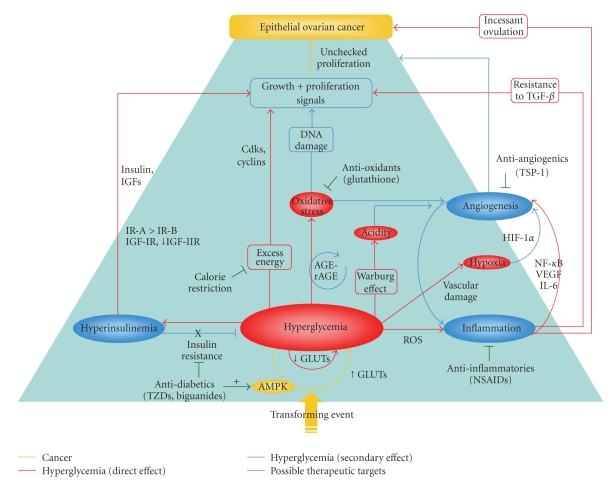
Summary diagram of factors hypothesized to link hyperglycemia to the development of epithelial ovarian cancer. Hyperglycemia, leading to hyperinsulinemia and inflammation, underlies the development of parallel pathologies affecting growth and death signaling, formation of reactive species, and angiogenesis. Together, these aberrant signals converge on a hyperproliferative phenotype that may promote or initiate the development of cancer. Possible therapeutic approaches, including the novel application of antidiabetic drugs, are shown in green. *Abbreviations**:*** TZDs, thiazolidinedoines; GLUTs, facilitative glucose transporters; ROS, reactive oxygen species; NSAIDs, nonsteroidal antiinflammatory drugs; AGE-RAGE, advanced glycation end product receptor complex; IR-A and IR-B, insulin receptor isoforms A and B; IGF(R), insulin-like growth factor (receptor); cdk, cyclin-dependant kinase; TSP-1, thrombospondin-1; HIF-1*α*, hypoxia-inducible factor alpha; NF-*κ*B, nuclear factor kappa B; VEGF, vascular endothelial growth factor.

An emerging view of cancer relies on an initiation-promotion paradigm that suggests a fundamental role of the tumour environment on cancer development. New data suggests that hyperglycemia may be a contributing factor to the onset and progression of EOC through a number of complex mechanisms (summarized in Figure 1). We propose that hyperglycemia has important effects on both the progression and somatic evolution of epithelial ovarian cancer. Altered glucose homeostasis is common in cancer patients, so antihyperglycemic therapies are applicable to even those who have normal blood sugar. Although there are a number of cellular mechanisms through which hyperglycemia may effect the promotion or initiation of ovarian cancer, there is almost no in vivo experimental data exploring the link between hyperglycemia and EOC. Further research in this area not only has applications in the development of cancer therapeutics, but also will provide new insights into EOC pathogenesis, early detection, and possible prevention.

## Figures and Tables

**Table 1:** Glucose transporter expression in ovarian and other cancers.

Facilitative Transporters: Class 1 GLUTs				

	Major site of expression	Expression in EOC [77–80]	Localization in EOC [77–80]	Expression in other cancers
GLUT-1	Fetal tissue, erythrocytes; widely distributed	Overexpressed in almost all invasive carcinomas; expression increases from benign to invasive tumours	Cell membrane, cytoplasm; more in membrane in more invasive; some studies say stronger closer to periphery; some say farther from tumour-stromal interface	Breast [82, 83], head, and neck [84], colorectal [85], prostate [86], pancreatic [87], cervical [88]
GLUT-2	Liver, pancreas	Negative	Unknown	Islet cell tumours [89], sarcoma [90]
GLUT-3	Brain	Conflicting: reported to be high in >90% of EOC tumours; also weak, homogenous expression in all ovarian tissue; also in ovarian tumours but not normal tissue	Cytoplasm and cell membrane	Lymphoma [91], head and neck [92], lung [93]
GLUT-4	Insulin-responsive tissues (skeletal muscle, heart, adipose tissue)	Conflicting: no expression in normal or malignant; also present in up to 84% in ovarian tumour cells	Unknown	Lung [94], breast [95]

Active Transporters: SGLTs.				

	Major site of expression	Expression in EOC	Localization in EOC	Expression in other cancers
SGLT1	Kidney and small intestine	Not investigated	Unknown	Breast [96], prostate [96], head and neck [97], pancreatic [98]
SGLT2	Kidney and small intestine	Not investigated	Unknown	No reports
SGLT3	Skeletal muscle and small intestine	Not investigated	Unknown	No reports
